# Fish occurrence in the middle Volga and upper Don regions (Russia)

**DOI:** 10.3897/BDJ.8.e54959

**Published:** 2020-10-08

**Authors:** Oleg Artaev, Alexander Ruchin, Victor Ivanchev, Elena Ivancheva, Vladimir Sarychev, Olga Moreva, Vyacheslav Mikheev, Dmirty Medvedev, Alexey Klevakin

**Affiliations:** 1 Papanin Institute for Biology of Inland Waters Russian Academy of Sciences, Borok, Russia Papanin Institute for Biology of Inland Waters Russian Academy of Sciences Borok Russia; 2 Joint Directorate of the Mordovia State Nature Reserve and National Park "Smolny", Saransk, Russia Joint Directorate of the Mordovia State Nature Reserve and National Park "Smolny" Saransk Russia; 3 Oka Nature Reserve, Ryazan, Russia Oka Nature Reserve Ryazan Russia; 4 Galichya Gora Reserve, Lipetsk, Russia Galichya Gora Reserve Lipetsk Russia; 5 Nizhny Novgorod branch of State Research Institute on Lake and River Fisheries, Nizhny Novgorod, Russia Nizhny Novgorod branch of State Research Institute on Lake and River Fisheries Nizhny Novgorod Russia; 6 Ulyanovsk State Pedagogical University, Ulyanovsk, Russia Ulyanovsk State Pedagogical University Ulyanovsk Russia; 7 Severtsov Institute of Ecology and Evolution, Russian Academy of Sciences, Moscow, Russia Severtsov Institute of Ecology and Evolution, Russian Academy of Sciences Moscow Russia

**Keywords:** Cephalaspidomorphi, Actinopterygii, database, Volga river, Don river

## Abstract

**Background:**

In ichthyological publications from both Russia as a whole, and from the study region, lack of data indicating the actual results of observations in a specific place all result in publication of a generalised analysis. Although our publications contain such data, they are, however, not convenient for users performing global analysis. The main purpose of publishing a database is to make our data available in the global biodiversity system to a wide range of users. Dataset represents a significant addition to the distribution of species in this area. The data can be used to analyse future changes in ichthyofauna, as well as to help the authorities to manage their territory more efficiently.

This publication describes a dataset that contains information on fish encounters in the Upper Don basin and the middle Volga (centre of the European part of Russia) over a 30-year period (1990-2020). The dataset contains information on 6400 occurrences of 394341 specimens of 56 species, 99.9% of specimens being identified to the species level. A total of 883 sites were studied, of which 253 were in lentic biotopes (lakes - 121, ponds - 123, backwater - 5, reservoir - 3, pit - 1), 630 were in lotic (rivers - 628, stream - 1, channel - 1). One collecting site has an average of 7.2 species (from 1-21 species per location). Only those species that form self-reproducing populations are given. The dataset is a compilation of data from several working author groups. All observations have precise geo-referencing with the names of water bodies (rivers, lakes etc.).

**New information:**

All presented data are published in the form of a database for the first time. Some data form the basis of previously-published works (3998 observations, 62%) and some are published for the first time (2402 observations, 38%). A large amount of data comes from small water bodies that have been neglected by previous researchers.

## Introduction

In recent years, the ichthyofauna of the rivers and reservoirs of many regions has changed significantly. This is due to a decrease in precipitation, the destruction of small river beds, the regulation of the flow of medium and large rivers, eutrophication, toxicity and thermification of water bodies and streams, water abstraction, peat extraction and light pollution (Ruban and Khodorevskaya 2011, Brüning et al. 2015, Boznak et al. 2019, Jimenez-Ojeda et al. 2019, Ling et al. 2017, Sowińska-Świerkosz and Kolejko 2019). All these processes lead to a reduction in diversity of indigenous species, disruptions in structure and functioning of freshwater ecosystems, communities and populations of fish species, to degradation and simplification of biota and to a reduction in number of optimal habitats ([Bibr B5780143][Bibr B5779519], Busarova et al. 2019). Alien species which, in some cases, have a significant impact on ichthyofauna of a particular reservoir and the region as a whole, are very important (Kirilenko and Shemonaev 2017, Brandner et al. 2018, Čolić et al. 2018, Ruchin et al. 2019). In the studied region, appearance of *Carassius gibelio, Rhynchocypris percnurus* or *Perccottus glenii* in an isolated reservoir led to the almost complete disappearance of previously-existing species (*Leucaspius delineatus*, *Carassius carassius*, *Misgurnus fossilis*, *Rutilus rutilus*) (Artaev and Ruchin 2017, Ruchin et al. 2016). In many administrative regions of Russia, due to various reasons associated with the negative state of the country's economy with the ensuing consequences, an inventory of the ichthyofauna has not been carried out since the 1990s. Our studies have shown that the distribution and abundance of many species differs significantly from the data of the last century (Ruchin et al. 2007, Ruchin et al. 2008, Ruchin et al. 2009, Artaev and Ruchin 2016, Ivanchev et al. 2013b, Sarycheva et al. 2014, Ivancheva et al. 2014).

The studied region is located at the centre of the European part of Russia, is densely populated and located in the immediate vicinity of Moscow, the administrative centre of Russia. Studies on the regional ichthyofauna have been carrying out quite intensively since the 1920s. We conducted a detailed analysis and generalisation of them in previous works (Artaev and Ruchin 2017, Ivanchev and Ivancheva 2010, Ivanchev et al. 2013a, Ruchin et al. 2016).

At the present stage of research, the main difference in this project is the study of a large number of different water bodies, including the smallest ones that were not represented in earlier studies, with detailed indication of species, habitats and dates. Previous efforts have usually presented only analyses and made certain conclusions on water bodies (Dushin 1967, Fedorov 1962, Fedorov 1970).

**Figure 1.**
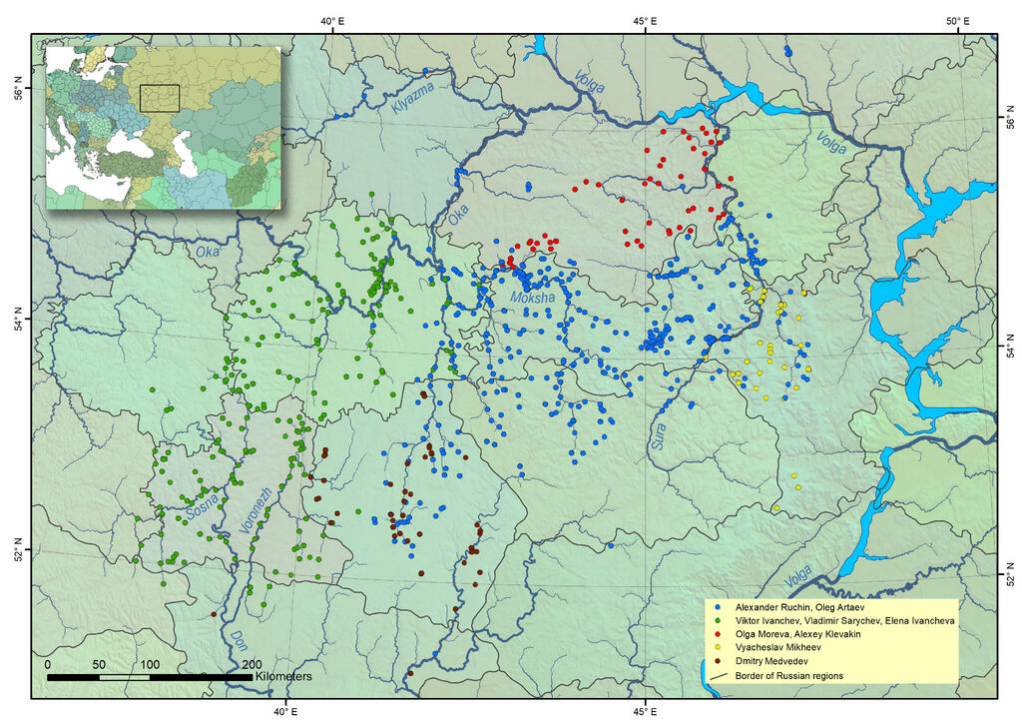
Collecting sites in the middle Volga and upper Don regions, explored by various working groups. Map was created in ArcGIS 10.8 software (www.esri.com).

Information from this dataset is the basis of a number of published monographs and articles (Artaev and Ruchin 2017, Ivanchev and Ivancheva 2010, Ivanchev et al. 2013a, Ruchin et al. 2016). It contains a compilation of data from several research groups (Fig. 1).

## Project description

### Title

Fish occurrence in middle Volga and upper Don regions (Russia)

## Sampling methods

### Study extent

The dataset contains information on 6400 occurrences (one species in a definite place at a definite time) of 394290 specimens encompasing 56 species made over the past 30 years (1990-2020). The study area is about 280000 km^2^.

### Sampling description

Fish were caught by various types of fishing gear (fry drag, seine nets, frame nets, gillnets with different mesh sizes, float and bottom fishing rods, spinning). The surveyed stream reaches were 200-500 m long and 3-10 m wide. The determination took place directly at the place of capture or fish were fixed in a 10% formalin and the determination was carried out in a laboratories (Mordovia State Nature Reserve, Oka Nature Reserve, Galichya Gora Nature Reserve and Nizhny Novgoros branch of the State Research Institute on Lake and River Fisheries). About 2-3% speciments was stored in the collection of the Mordovia State Nature Reserve.

### Quality control

Each observation contained fundamental information, such as location (coordinates), date, name of water bodies, name of observer and name of identifier. A large part of the coordinates was determined directly on site with the help of a GPS device. In other cases, Google Maps (2020) were used. The names of water bodies were given from topographic maps (Anonymous 2020) or determined according to local residents. Species were determined by Reshetnikov (2010), Reshetnikov et al. (2003) and Kottelat and Freyhof 2007), considering recent taxonomic compilations proposed by Fricke et al. 2020.

## Geographic coverage

### Description

The dataset contains data on fish occurrences within the territory of 15 Russian regions: the Republic of Mordovia, the Chuvash Republic, the Republic of Mari-El, Vladimir, Ryazan, Tula, Tambov, Penza, Ulyanovsk, Oryol, Voronezh, Kursk, Saratov, Ulyanovsk and Nizhny Novgorod Regions. The main observations are concentrated in the Don basin above Voronezh, in the middle reaches of the Oka basin, the Moksha basin and the middle and lower reaches of the Sura river (Fig. 1).

The study area is located on the East European Plain. In the east, there is Volga Upland with maximum heights of up to 350 m above sea level and in the West, there is Central Russian Upland (up to 300 m above sea level). Between, there is the Oksko-Don Plain (up to 180 m above sea level). Minimum heights of the study area are the Don in Voronezh (90 m) and the Volga in Nizhny Novgorod (70 m). The territory is located in the temperate climate zone. The total duration of the period with average daily air temperature below feezing point is 140-150 days per year. The study area is divided into two different basins - the Black Sea basin (Don River watershed) and the basin of the inland Caspian Sea (Volga River watershed). All rivers of the region are typically lowland and belong to the East European type. Its main characteristic is seasonal flow, such as distinct spring floods, relatively low summer and winter water levels, as well as increased run-off in autumn. In summer, sometimes the water level rises as a result of heavy rain. The rivers have a mixed supply, snowmelt, which accounts for most of the annual run-off, as well as from precipitation and groundwater. The vast majority of the lakes are oxbows. Most of them are located in the Oka-Don lowland. In the north (mainly in the Nizhny Novgorod Region in the Piana and Tesha watershed), there is a small number of karst lakes. There are also a small number of aeolian and suffosion lakes. Ponds were formed on many rivers after the construction of dams (Kuznetsov 1974, Okorokov et al. 2003, Savrasova 1998, Yamashkin 1998, Krivtsov 2008).

### Coordinates

51.89 and 57.421 Latitude; 37.441 and 48.999 Longitude.

## Taxonomic coverage

### Description

Taxonomic diversity of the study area is represented by 56 species (55 ray-finned fishes and one lamprey species) belonging to 16 families of nine orders. Given the scale of focused research on fauna, this is an almost exhaustive list of species that form natural self-reproducing populations.

## Traits coverage

### Data coverage of traits

PLEASE FILL IN TRAIT INFORMATION HERE

## Temporal coverage

### Notes

May 1990 through to May 2020

## Usage rights

### Use license

Creative Commons Public Domain Waiver (CC-Zero)

### IP rights notes

This work is licensed under a Creative Commons Attribution Non-Commercial (CC-BY-NC) 4.0 Licence.

## Data resources

### Data package title

Fish occurrence in middle Volga and upper Don regions (Russia)

### Resource link

https://www.gbif.org/dataset/e79ec95a-a90f-4f99-819c-8e4ec58e6bd3

### Alternative identifiers

https://doi.org/10.15468/ru9p9j

### Number of data sets

1

### Data set 1.

#### Data set name

Fish occurrence in middle Volga and upper Don regions (Russia)

#### Data format

Darwin Core

#### Number of columns

16

#### Description

Published and unpublished author's data on fish occurrence in middle Volga and upper Don regions (Russia)

**Data set 1.** 

Column label	Column description
occurrenceID	The Globally Unique Identifier number for the recored
basisOfRecord	The specific nature of the data record: HumanObservation
eventDate	date format as YYYY-MM-DD
year	Year of the event was recorded
month	The month of the event was recorded
day	The integer day of the month on which the Event occurred
scientificName	The full scientific name including the genus name and the lowest level of taxonomic rank with the authority
kingdom	The full scientific name of the kingdom in which the taxon is classified
decimalLatitude	The geographic latitude of location in decimal degrees
decimalLongitude	The geographic longitude of location in decimal degrees
geodeticDatum	The geodetic datum for coordinates: WGS84
country	The name of the country (Russia)
identifiedBy	A list of names of people, who assigned the Taxon to the subject
recordedBy	A person or group responsible for recording the original Occurrence
associatedReferences	Bibliographic reference of literature associated with the Occurrence
waterBody	The name of the water body in which the Location occurs

## Additional information

This dataset provides reliable records that contribute to increasing knowledge on the distribution of fish species on middle Volga and upper Don regions. The dataset contains information on 6400 occurrences of 56 species that form self-reproducing populations (Artaev et al. 2020, Table 1).

## Figures and Tables

**Table 1.** Taxonomic composition of the dataset, number of observations and individuals. Taxonomy follows Fricke et al. (2020).

Taxa	Number of observations	Number of speciments
** Cephalaspidomorphi **		
** Petromyzontiformes **		
** Petromyzontidae **		
*Eudontomyzon mariae* (Berg, 1931)	18	23
** Actinopterygii **		
** Acipenseriformes **		
** Acipenseridae **		
*Acipenser ruthenus* Linnaeus, 1758	12	32
** Cypriniformes **		
** Acheilognathidae **		
*Rhodeus amarus* (Bloch, 1782)	248	47860
** Cobitidae **		
*Cobitis melanoleuca* Nichols, 1925	95	531
*Cobitis* sp.	11	92
*Cobitis taenia* Linnaeus, 1758	205	1262
*Cobitis tanaitica* Băcescu & Mayer, 1969	2	3
*Misgurnus fossilis* (Linnaeus, 1758)	37	232
*Sabanejewia aurata* (De Filippi, 1863)	3	17
*Sabanejewia baltica* Witkowski, 1994	22	216
** Cyprinidae **		
*Carassius carassius* (Linnaeus, 1758)	57	917
*Carassius gibelio* (Bloch, 1782)	202	4771
*Cyprinus carpio* Linnaeus, 1758	36	2628
** Gobionidae **		
*Gobio brevicirris* Fowler, 1976	102	7804
*Gobio volgensis* Vasil’eva, Mendel, Vasil’ev, Lusk & Lusková, 2008	307	10066
*Pseudorasbora parva* (Temminck & Schlegel 1846)	13	193
*Romanogobio albipinnatus* (Lukasch, 1933)	102	8904
** Leuciscidae **		
*Abramis ballerus* (Linnaeus, 1758)	23	2372
*Abramis brama* (Linnaeus, 1758)	164	19154
*Alburnoides rossicus* Berg, 1924	94	3035
*Alburnus alburnus* (Linnaeus, 1758)	446	50049
*Alburnus chalcoides* (Güldenstädt 1772)	1	1
*Ballerus sap*a (Pallas, 1814)	27	129
*Blicca bjoerkna* (Linnaeus, 1758)	193	21905
*Chondrostoma variabile* Yakovlev, 1870	48	2581
*Leucaspius delineatus* (Heckel, 1843)	296	29988
*Leuciscus aspius* (Linnaeus, 1758)	84	522
*Leuciscus danilewskii* (Kessler 1877)	20	494
*Leuciscus idus* (Linnaeus, 1758)	175	1348
*Leuciscus leuciscus* (Linnaeus, 1758)	331	20571
*Leuciscus* sp.	5	214
*Pelecus cultratus* (Linnaeus, 1758)	12	79
*Phoxinus phoxinus* (Linnaeus, 1758)	82	21391
*Rhynchocypris percnurus* (Pallas, 1814)	39	3744
*Rutilus frisii* (Nordmann, 1840)	6	1225
*Rutilus rutilus* (Linnaeus, 1758)	580	69016
*Scardinius erythrophthalmus* (Linnaeus, 1758)	158	7194
*Squalius cephalus* (Linnaeus, 1758)	315	10655
*Vimba vimba* (Linnaeus 1758)	34	1334
** Nemacheilidae **		
*Barbatula barbatula* (Linnaeus, 1758)	298	3494
** Tincidae **		
*Tinca tinca* (Linnaeus, 1758)	48	202
** Esociformes **		
** Esocidae **		
*Esox lucius* Linnaeus 1758	424	4486
** Gadiformes **		
** Lotidae **		
*Lota lota* (Linnaeus, 1758)	78	183
** Perciformes **		
** Gobiidae **		
*Benthophilus stellatus* (Sauvage 1874)	2	15
*Neogobius fluviatilis* (Pallas, 1814)	48	862
*Neogobius melanostomus* (Pallas, 1814)	7	110
*Proterorhinus marmoratus* (Pallas 1814)	36	826
** Odontobutidae **		
*Perccottus glenii* Dybowski, 1877	175	8683
** Percidae **		
*Gymnocephalus acerina* (Gmelin, 1789)	4	336
*Gymnocephalus cernua* (Linnaeus, 1758)	171	5685
*Perca fluviatilis* Linnaeus, 1758	449	16634
*Sander lucioperca* (Linnaeus, 1758)	29	217
*Sander volgensis* (Gmelin, 1789)	1	1
** Salmoniformes **		
** Salmonidae **		
*Salmo trutta* Linnaeus, 1758	1	1
** Scorpaeniformes **		
** Cottidae **		
*Cottus koshewnikowi* Gratzianov, 1907	6	24
** Siluriformes **		
** Siluridae **		
*Silurus glanis* Linnaeus, 1758	18	30
